# Increasing the measurable frequency range of magnetic field oscillations with off‐resonance spin‐lock preparation pulses

**DOI:** 10.1002/mrm.70116

**Published:** 2025-10-08

**Authors:** Fróði Gregersen, Teresa Cunha, Axel Thielscher, Lars G. Hanson

**Affiliations:** ^1^ Section for Magnetic Resonance, DTU Health Tech Technical University of Denmark Kgs. Lyngby Denmark; ^2^ Danish Research Centre for Magnetic Resonance, Department of Radiology and Nuclear Medicine Copenhagen University Hospital—Amager and Hvidovre Copenhagen Denmark

**Keywords:** brain stimulation, neuronal currents, spin‐lock, two‐photon MRI

## Abstract

**Purpose:**

Oscillatory magnetic fields—arising from, e.g., neuronal currents or induced currents during brain stimulation—can be measured with spin‐lock preparation pulses, where the frequency of the measured field matches the amplitude of the spin‐lock pulse. The upper frequency limit is thus determined by either the maximum RF amplitude or SAR restrictions. This study aimed to extend the measurable frequency range.

**Theory and Methods:**

We outline theoretically how off‐resonance preparation pulses lead to an extended measurable frequency range of oscillating fields and how to tune them to the frequency of interest, and we discuss how this is related to the theory of two‐photon excitation. We use the MR simulator software KomaMRI to simulate our proposed sequence and compare it to phantom measurements, where the oscillatory magnetic field is created from a current in a cable loop around a phantom.

**Results:**

The simulations and measurements were consistent, showing that the RF amplitude can be decreased when the off‐resonance detuning is increased while being sensitive to the same frequency of the oscillating field. We additionally observed that the effects of the RF inhomogeneities on the measured signal were reduced as the detuning increased.

**Conclusion:**

We have shown that the measurable frequency range of magnetic field oscillations can be increased using off‐resonance preparation pulses, albeit with a lower sensitivity. We also observed reduced effects from RF inhomogeneities, which can potentially improve neuronal current detection at low frequencies as well.

## 1 INTRODUCTION

Obtaining a localized, direct, noninvasive measurement of brain activity has been a long‐standing goal in MRI research. One of the most promising and studied mechanisms is to measure the influence of the neuronal current‐induced magnetic field on the MR signal. Most techniques have focused on measuring either the phase shift or dephasing caused by local magnetic field oscillations.^1^ Using published methods, the accumulated phase shift in a voxel can be made sensitive to coherent temporal field variation, e.g., reflected in electroencephalography that results from synchronized neuronal activity. In contrast, spatial field variation within a voxel causes phase cancellation that can also result from incoherent field fluctuations and variation in temporal field dynamics within a voxel.^2^, ^3^ These dephasing effects that may result from neuronal activation can be reflected in the MR signal amplitude. Amplitude measurements are consequently more robust correlates of activation, but are also much less sensitive than optimized phase‐sensitive measurements when there is spatial and temporal coherence within a voxel.

In 2008, Witzel et al.^4^ proposed to use a spin‐lock preparation pulse to measure the reduction in the $T_{1\rho }$ relaxation time caused by oscillatory magnetic fields originating from neuronal currents. The method was named Stimulus‐Induced Rotary Saturation (SIRS). For nuclei with a gyromagnetic ratio of γ, it is sensitive to oscillations at the Larmor frequency in the rotating frame ($\gamma B_{1}$), and can be tuned by adjusting the amplitude $B_{1}$ of the spin‐lock pulse. Importantly, it can be used for measuring neuronal currents, where there is uncertainty about the spatial and temporal phase of the field oscillations, as it is tolerant to spatial and temporal incoherence. Later, Jiang et al.^5^ suggested a modification to the sequence with greatly increased sensitivity but with the downside of being sensitive to spatial phase cancellation. Rather than measuring a saturation effect, it measures an excitation away from the magnetization trajectories characteristic of undisturbed precession around a spin‐lock field oscillating at the Larmor frequency corresponding to the static $B_{0}$ field. Only additional fields that oscillate near the Larmor frequency in the rotating frame, $\gamma B_{1}$, cause such excitation. Together with back‐rotation of the magnetization to a near‐longitudinal direction, as introduced by Jiang et al.,^5^ or spoiling transverse magnetization followed by a $90^{\circ }$ excitation pulse introduced by Truong et al.,^6^ this gives an advantageous linear signal dependence for small excitation angles compared to SIRS, and the physiological noise sensitivity is low since subtraction of high‐intensity images is avoided. The method of measuring the excitation away from the spin‐lock axis, rather than the saturation of the magnetization along the spin‐lock axis, has been termed rotary excitation (REX).^7^

Measuring oscillatory fields with either SIRS or REX has, to our knowledge, only been discussed in the context of neuronal currents, where the relevant frequency range of oscillatory activity is generally considered to be within 1–100 Hz,^6^, ^7^, ^8^, ^9^, ^10^ of detecting epileptic activity at 120 Hz and 240 Hz,^11^ and of measuring cardiac fields at sub 200 Hz.^12^ However, there are several brain stimulation and emerging electric‐field‐based cancer therapies that could benefit from mapping the current density by measuring the current‐induced magnetic fields over a broader frequency range up to several kHz and beyond. Examples of these techniques are transcranial electric stimulation (0–640 Hz),^13^ deep brain stimulation (60–200 Hz),^14^ temporal interference stimulation (kHz range),^15^ and tumor‐treating fields (100–300 kHz).^16^ Other techniques have previously been used to map the current‐induced magnetic fields such as Magnetic Resonance Current Density Imaging (MRCDI) (0–20 Hz),^17^ AC‐CDI (100–1 000 Hz)^18^ and RF‐CDI (at the Larmor frequency),^19^ where MRCDI is the only technique that has been used to map current‐induced magnetic fields in the human head.^20^, ^21^, ^22^, ^23^, ^24^ However, there is a discrepancy between the frequencies of the applications of interest and what current methods can measure. Since the Larmor frequency in the rotating frame can be tuned by adjusting the $B_{1}$ amplitude, spin‐lock preparation is a good candidate for extending the measurable frequencies of oscillating fields beyond the capability of current methods. However, there is also a practical upper limit for tuning the Larmor frequency in the rotating frame given by either the maximum $B_{1}$ amplitude or RF power deposition (SAR) causing tissue heating. Liimatainen et al.^25^ explored using off‐resonance spin‐lock pulses to study the relaxation along fictitious fields for novel MR contrasts and noted the reduction of RF power compared to on‐resonance pulses.

Here, we explore using off‐resonance preparation pulses to increase the Larmor frequency associated with the spin‐lock pulse without increasing the $B_{1}$ amplitude or SAR. A preliminary and partial account of this study was presented as a conference contribution.^26^ We first show how off‐resonance pulses can be used to increase the frequency of sensitivity without increasing the $B_{1}$ amplitude. We then perform Bloch simulations of our proposed method as well as phantom measurements for validation. Since magnetic fields from injected currents vary slowly over space and are controlled in time, phase cancellation is less of an issue than for neuronal currents. We will therefore only focus on REX, which has higher sensitivity than SIRS, but the concepts presented here can easily be transferred to SIRS.

## 2 THEORY

**FIGURE 1:**
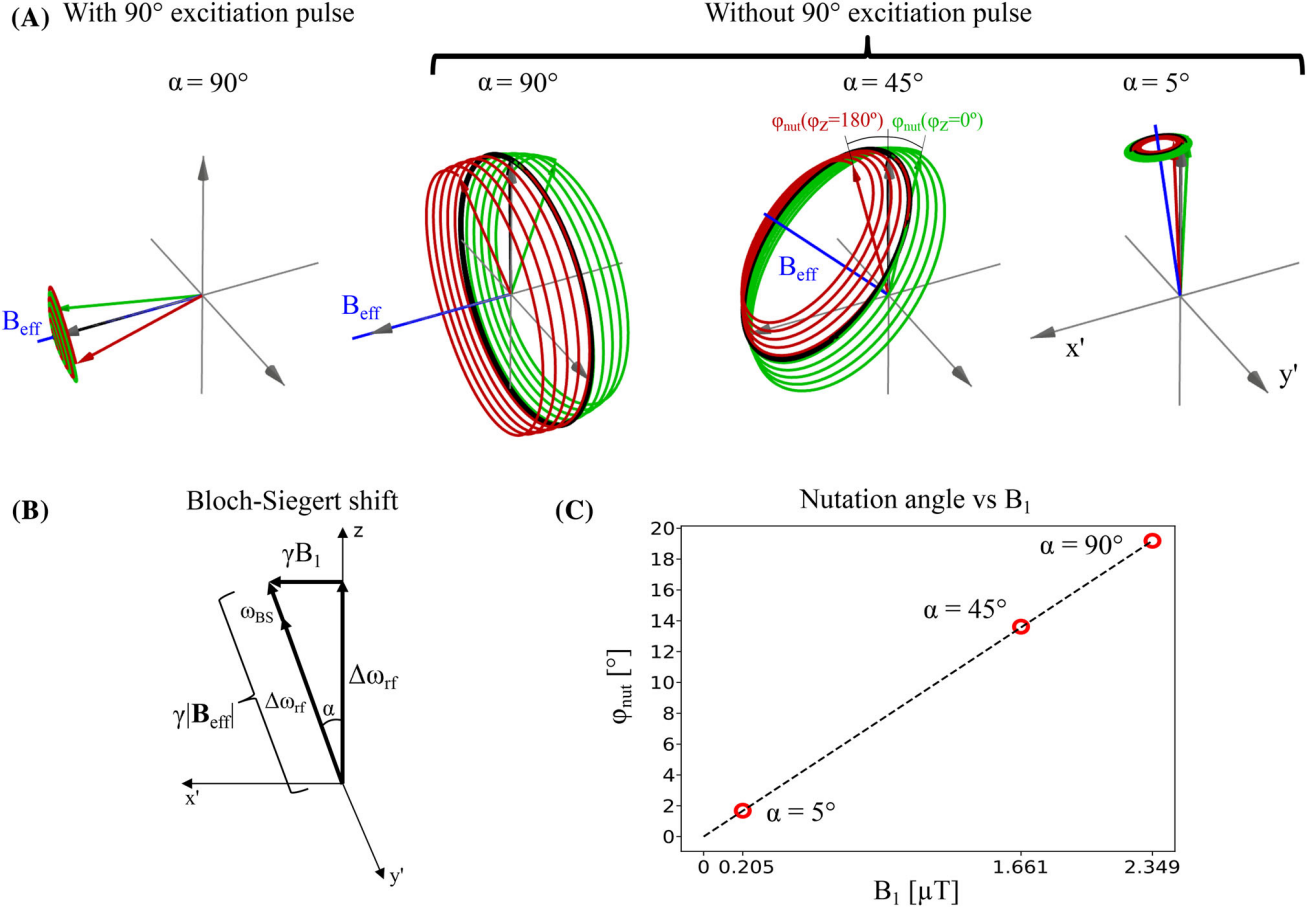
(A) Simulation of the REX effect with and without a preceding $90^{\circ }$ excitation pulse. The REX effect, depicted by the red and green vectors and their traces, is caused by a field oscillating at $\omega_{z}/2\pi$ = 100 Hz, $B_{z}$ = 50 nT, a spin‐lock time ($T_{\text{SL}}$) = 50 ms, no relaxation, and with varying off‐resonance frequencies reflected in α that is the angle between $B_{0}$ and the spin‐lock axis. $\gamma |B_{\text{eff}}|$ = 100 Hz in all four cases. The red and green traces are caused by $180^{\circ }$ phase‐shifted versions of $B_{z}$ (see Figure 2). (B) A vector illustration of the effective (spin‐lock) field vector and the Bloch‐Siegert shift correction. (C) The REX nutation angle caused by a $B_{z}$‐oscillation is linearly proportional to the amplitude of the spin‐lock pulse when relaxation is ignored. The simulation parameters are as listed for Figure 1A.

In the frame rotating at frequency $\omega_{\text{rot}}=\omega_{\text{rf}}$ and at resonance ($\omega_{\text{rf}}=\gamma B_{0}$), a constant $B_{1}$ field—the spin‐lock field—takes the role of $B_{0}$ in the laboratory frame and effectively lowers the Larmor frequency to $\gamma B_{1}$, referred to as the Larmor frequency in the rotating frame. A field oscillating in the z‐direction ($B_{z}$) at the frequency $\gamma B_{1}$ will thus result in the REX effect as shown by the red and green vectors and traces in the first image in Figure 1A (see Section 3.1 for details on the numerical simulations). A $90^{\circ }$ excitation pulse is usually applied before the spin‐lock pulse to align the net magnetization (M) parallel to the spin‐lock axis. However, excitation is not necessary as M is precessing around $B_{1}$ in the rotating frame independently of M's orientation. The REX effect caused by $B_{z}$ results in a nutation away from the precession around $B_{1}$ as seen in the second image in Figure 1A, where no $90^{\circ }$ excitation pulse was applied before the spin‐lock pulse.

With an off‐resonance spin‐lock pulse, the Larmor frequency in the rotating frame is $\gamma |B_{\text{eff}}|$, where $\left|B_{\text{eff}}\right|$ is the amplitude of the effective field vector in the rotating frame. The Larmor frequency in the rotating frame for an off‐resonance spin‐lock pulse and the frequency of $B_{z}(\omega_{z}$) required for an excitation becomes

(1) $$\omega_{z}=\gamma |B_{\text{eff}}|=\gamma \sqrt{\Delta \omega_{\text{rf}}^{2}+{\left(\gamma B_{1}\right)}^{2}}$$

where $\Delta \omega_{\text{rf}}$ is the detuning of the spin‐lock pulse, $\Delta \omega_{\text{rf}}\equiv \omega_{\text{rf}}-\omega_{0}$ with $\omega_{0}\equiv \gamma B_{0}$. It is therefore possible to increase the Larmor frequency in the rotating frame without increasing $B_{1}$ by introducing an off‐resonance spin‐lock pulse. In the third and fourth images in Figure 1A, the REX effect is shown with two off‐resonance frequencies reflected in two values of the angle α between $B_{\text{eff}}$ and the z‐axis, α = $45^{\circ }$ and $5^{\circ }$.

**FIGURE 2:**
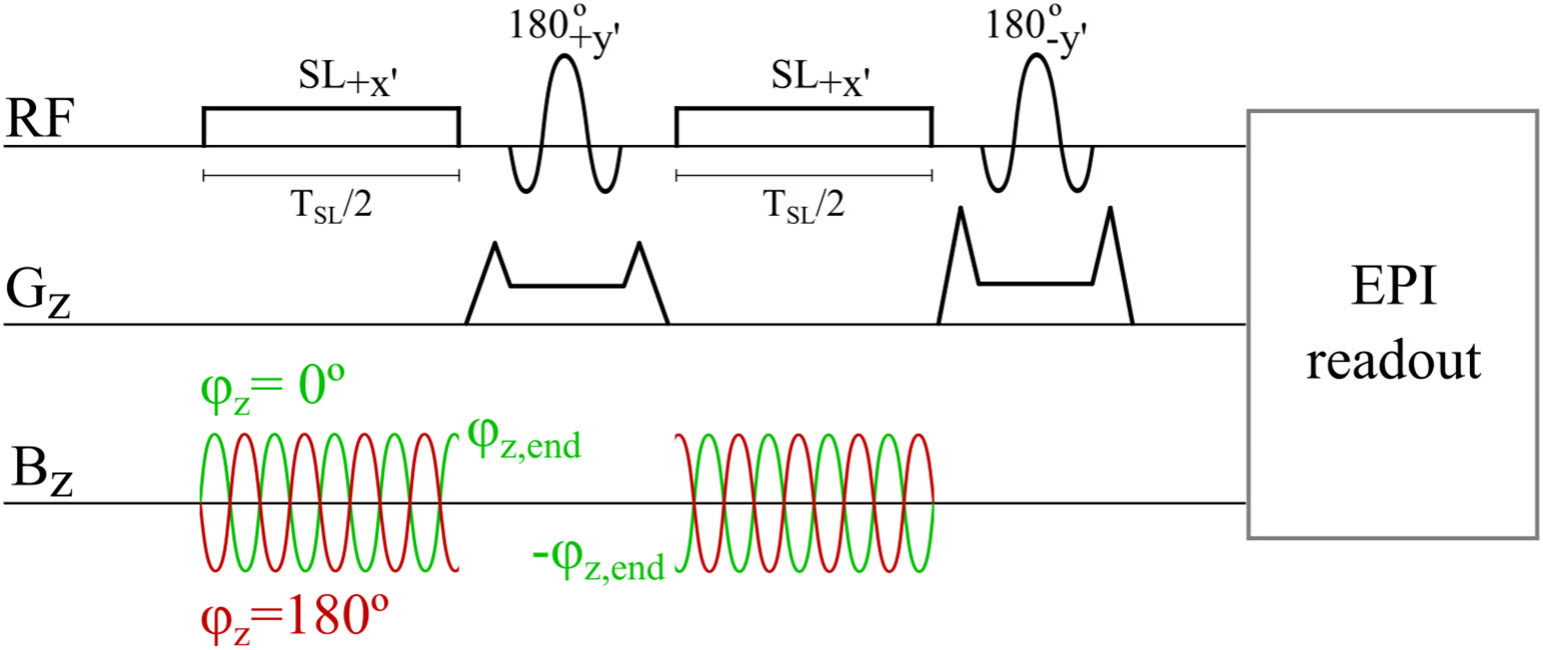
Diagram of the sequence used in the second simulation and the measurements. Encoding happens during the spin‐lock pulses, and $B_{z}$ is the oscillating field resulting in the REX effect. The first $180^{\circ }$ pulse reduces inhomogeneity effects, and the second pulse crushes out‐of‐slice excitation. An anti‐symmetry in the phase of $B_{z}$ between the first and the second encoding period is necessary to continue the REX effect as depicted in the figure.

Figure 1B,C is introduced in the Discussion that relates the theory of rotary excitation to multi‐photon excitation.

## 3 METHODS

### 3.1 Numerical simulations

The MR simulator KomaMRI^27^ was used to integrate the Bloch equations. Two sets of simulations were performed. The first simulations provided the effect of a $B_{z}$ field on the magnetization during a spin‐lock pulse with and without a $90^{\circ }$ excitation pulse. The spin‐lock pulse was simulated with varying off‐resonance frequency, and $B_{1}$ was adjusted to maintain the Larmor frequency $\gamma |B_{\text{eff}}|$ in the rotating frame at 100 Hz. These simulations were performed to visualize the REX effect caused by $B_{z}$ for a constant amplitude of $B_{\text{eff}}$ and varying angles with respect to the z‐axis. The simulations are presented in Figure 1 and are used to illustrate the points discussed in the theory section. Relaxation was ignored.

The second part simulates the sequence as shown in Figure 2, discussed in Section 3.2 below, and used in the imaging experiment. The sinusoidal $B_{z}$ field was simulated with $\varphi_{z}=0^{\circ }$ and $\varphi_{z}=180^{\circ }$, and the $B_{z}$‐induced signal ($S_{Bz}$) was calculated as discussed in Section 3.4. All simulations were performed with α = $90^{\circ }$, $45^{\circ }$, and $5^{\circ }$. The relaxation parameters for the phantom used in the experiment were also used in the simulations (T1 = 540 ms, T2 = 50 ms). The relaxation times were estimated with an IR‐EPI sequence with varying TI and long TR and a SE‐EPI sequence with varying TE and long TR, respectively.

### 3.2 Sequence

The sequence used in the experiments is presented in Figure 2. REX caused by $B_{z}$ occurs during the two spin‐lock pulses. The combination of the two spin‐lock pulses and the first $180^{\circ }$ pulse compensates for $B_{1}$ field inhomogeneities present during the spin‐lock pulses and for $B_{0}$ inhomogeneities. This is similar to the composite spin‐lock pulse introduced by Zeng et al.,^28^ but with a different phase combination of the RF pulses. The second $180^{\circ }$ RF pulse crushes out‐of‐slice excitation and performs the back‐rotation of the magnetization from negative near‐longitudinal to the positive near‐longitudinal axis before imaging. In the imaging experiment, an EPI readout was used while no imaging gradients were simulated.

### 3.3 Imaging experiment

The experiments were performed using a 3T clinical MR scanner (MAGNETOM Prisma, Siemens Healthineers, Erlangen, Germany). To generate $B_{z}$ in the experiment, a current with a sinusoidal waveform was passed through a cable wrapped three times around the FBIRN spherical agar gel phantom,^29^ thus forming a planar coil. The cable was made from conductive silicone rubber with a square cross section of 3 × 3 mm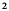 and a conductivity of 29.4 S/m to effectively eliminate interference with the RF field.^30^ The cable was connected to a Biopac MECMRI‐1 cable that was again connected to a Biopac MRIRFIF pi filter (BIOPAC Systems, Goleta, USA) at the Faraday cage wall. A current‐controlled neurostimulator (DC‐STIMULATOR MR, NeuroCare Group GmbH, Germany) outside of the scanner room was connected to the pi filer. The stimulator supplied a 4 mA baseline‐to‐peak current, resulting in a magnetic field of approximately 50 nT in the ROI delineated in Figure 4 was calculated using the Biot‐Savart law for the reconstructed cable path imaged with a UTE sequence.^22^ The current source was controlled by an Arduino microcontroller (Arduino.cc, Chiasso, Switzerland) that was programmed to supply the desired current. A trigger signal was received from the scanner at the start of each spin‐lock pulse. This was used to synchronize the Arduino with the MR sequence. $B_{z}$ was only applied during the spin‐lock pulses.

#### 3.3.1 Experiment 1: Verifying resonance

In the first experiment, the aim was to verify the resonance conditions according to Equation (1) by varying $\omega_{z}$. The Larmor frequency in the rotating frame, defined by $\gamma |B_{\text{eff}}|$, was kept at 100 Hz. The measurements were performed three times with α = $90^{\circ }$, $45^{\circ }$, and $5^{\circ }$. $\omega_{z}/2\pi$ was varied from 50 Hz to 150 Hz in steps of 10 Hz. Since the full‐width at half‐maximum of the signal frequency distribution for α = $90^{\circ }$ is smaller than for α = $45^{\circ }$ and $5^{\circ }$, additional measurements were conducted with $\omega_{z}/2\pi$ varying from 75 Hz to 125 Hz in steps of 10 Hz. The values of $B_{1}$ and $\Delta \omega_{\text{rf}}$ were calculated for a desired α and $\gamma |B_{\text{eff}}|$ according to Figure 1B. The other sequence parameters were $T_{R}$ = 166 ms, readout bandwidth per pixel = 2170 Hz, spin‐lock duration $T_{\text{SL}}$ = 80 ms (see Figure 2), time from start of EPI readout to center of k‐space = 33.4 ms, in‐plane resolution = (5 mm)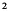, 1 slice with 5 mm slice thickness, (320 mm)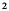 FOV, and 200 measurement repetitions.

#### 3.3.2 Experiment 2: Spin‐lock duration

The second experiment aimed to measure the relationship between $T_{\text{SL}}$ and the signal strength with $\omega_{z}/2\pi$ on resonance (100 Hz). The signal strength reflects a trade‐off between $T_{\text{SL}}$ and the relaxation rates and varies for different values of α. This experiment was also performed with alpha = $90^{\circ }$, $45^{\circ }$, and $5^{\circ }$. $T_{\text{SL}}$ was varied from 20 ms to 200 ms in steps of 20 ms, and $T_{R}$ varied from 106 ms to 286 ms with 20 ms steps. The other parameters were the same as in experiment 1.

### 3.4 Post processing

In all experiments, one slice was imaged repeatedly while alternating the phase of the cable current ($\varphi_{z}$, see Figure 2) between $0^{\circ }$ and $180^{\circ }$. The influence of $B_{z}$ on the measured signal is thus isolated from other signal sources as

(2) $$S_{Bz}=\overset{N/2}{\underset{i=1}{\sum }}|I_{\text{even}}(i)-I_{\text{odd}}(i)|$$

where N is the number of repetitions and $I_{\text{even}}$, $I_{\text{odd}}$ are the complex images from even and odd numbered repetitions.

The local Larmor frequency in the rotating frame in each voxel is determined by the $B_{0}$ and $B_{1}$ inhomogeneities. Adding the inhomogeneities, Equation (1) becomes

(3) $$\omega_{z}(r)=\gamma \sqrt{{\left(\Delta \omega_{\text{rf}}+\gamma \Delta B_{0}(r)\right)}^{2}+{\left(\gamma B_{1}B_{1,\text{rel}}(r)\right)}^{2}}$$

where $\Delta B_{0}$ is the measured local $B_{0}$ offset caused by field inhomogeneity, $B_{1}$ is the nominal $B_{1}$ field, $B_{1,\mathrm{rel}}$ is the measured relative $B_{1}$ field, and r denotes the voxel position.

We measured $B_{0}$ and $B_{1}$ maps with the phantom and wire loop in place, using the double $T_{E}$ and double angle^31^ methods, respectively.

**FIGURE 3:**
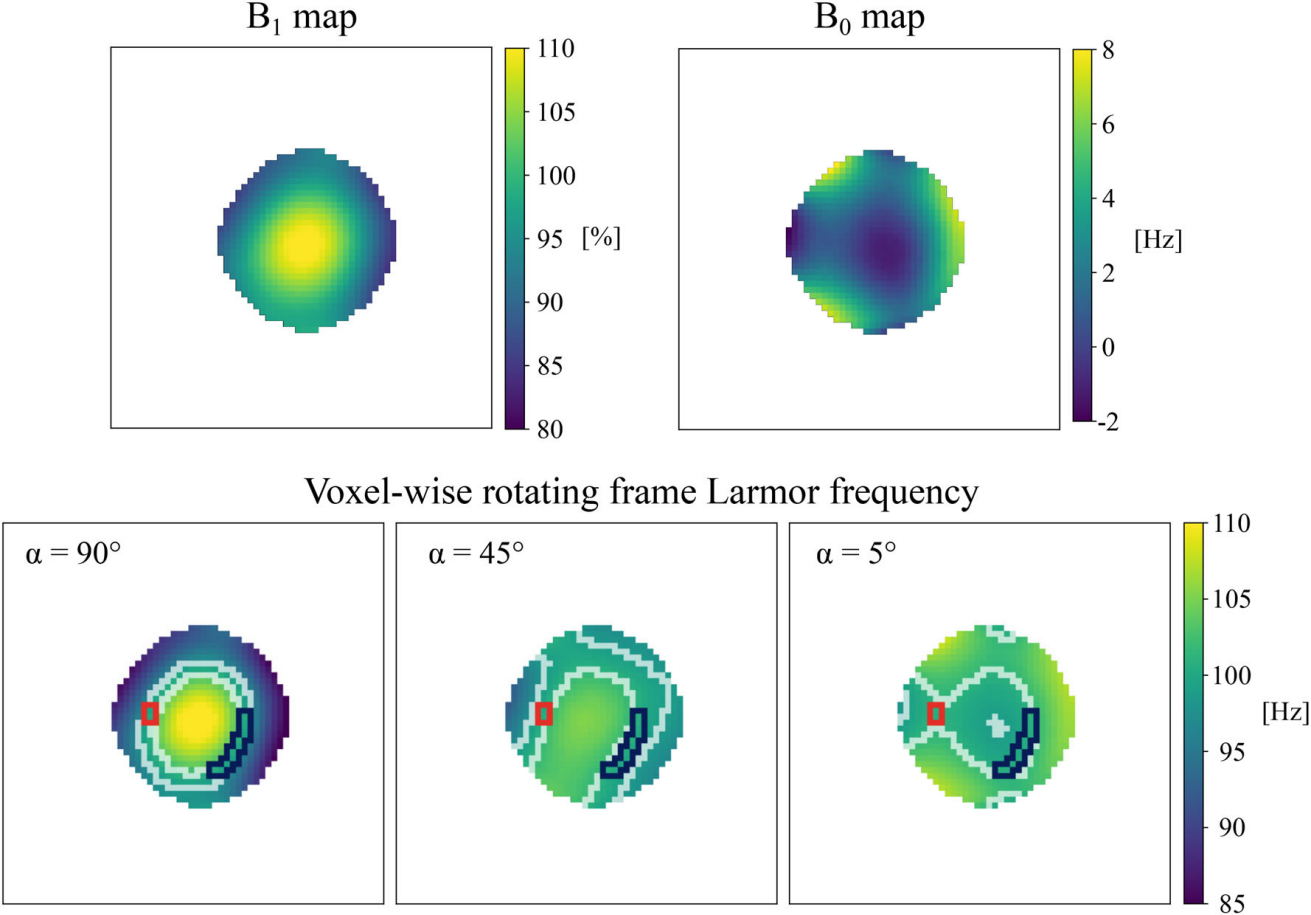
$B_{0}$ and $B_{1}$ field maps (top) used to calculate the voxel‐wise Larmor frequency in the rotating frame (bottom). The white contours indicate the regions where the Larmor frequency in the rotating frame is 100 ± 1 Hz, and the blue and red contours indicate the overlapping regions where the sequence is highly sensitive to field oscillation at this frequency for all the values of the flip angle α. The data from the larger cluster (blue contour) were compared to simulations (see Figure 4).

Since the measurement data varies spatially due to field inhomogeneities, we chose a region of interest (ROI) as a continuous region where the local Larmor frequency $\gamma B_{\text{eff}}$ was 100 ± 1 Hz for all three α values (Figure 3). To compare the simulated and measured data, all signals were normalized to the on‐resonance measurement in experiment 1 with $\alpha =90^{\circ }$.

## 4 RESULTS

### 4.1 Influence of inhomogeneities

The $B_{0}$ and $B_{1}$ inhomogeneities affect the local Larmor frequency in the rotating frame differently for different flip angles α (Figure 3). For small α, the $B_{0}$ inhomogeneity affects the local Larmor frequency $\gamma B_{\text{eff}}$ most, resulting in the similarity of the variation seen in the $\Delta B_{0}$ and the Larmor frequency maps. For large α, the Larmor frequency maps resemble the $B_{1}$ maps most.

**FIGURE 4:**
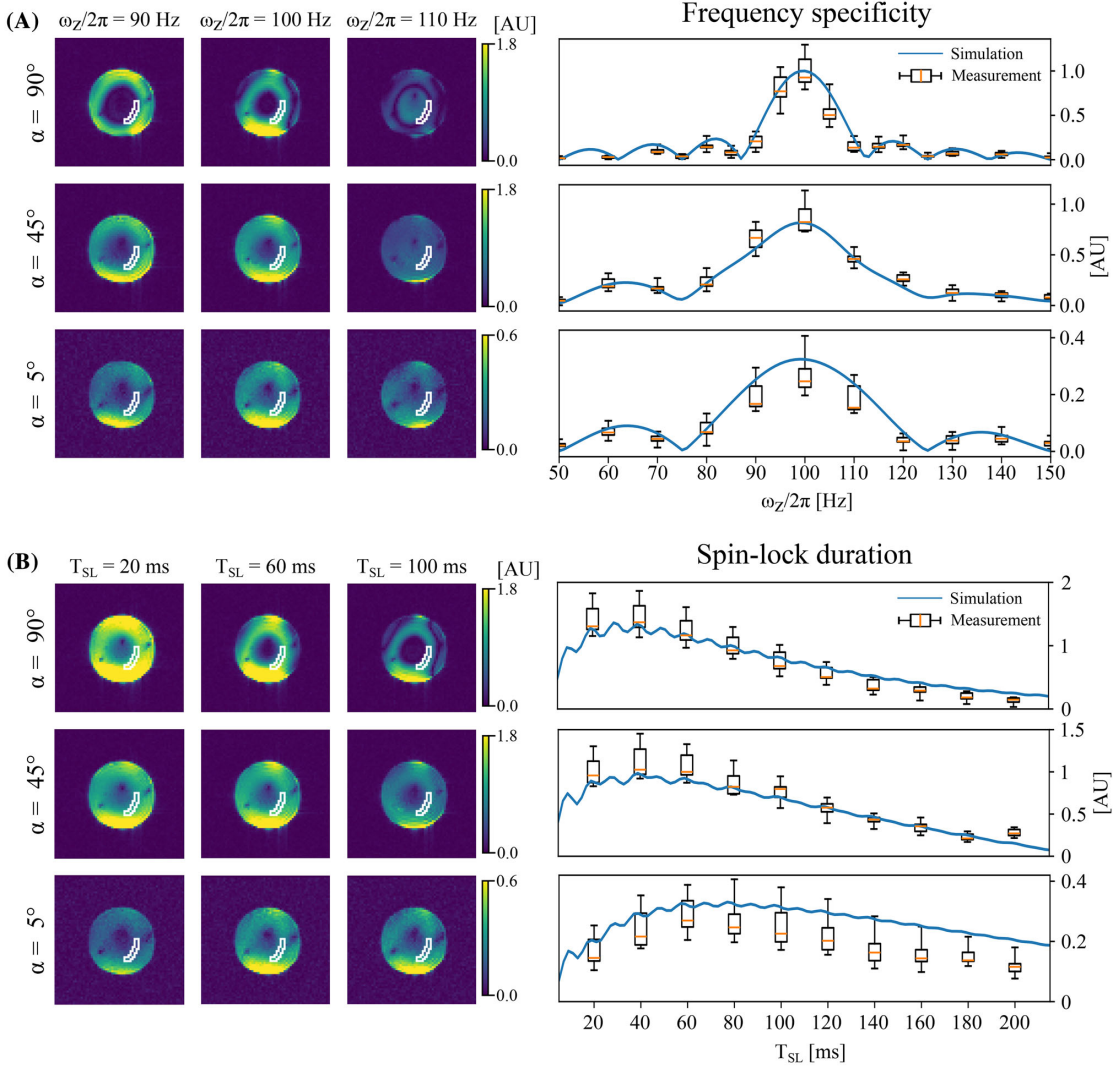
Results for the measured and simulated magnetic field‐induced signal $S_{Bz}$ as defined in Equation (3). (A) Simulation and measurements of frequency specificity by varying $\omega_{z}$. To the left are the measured images for the three flip angles α with current oscillation frequency $\omega_{z}/2\pi$ = 90, 100, and 110 Hz. Due to field inhomogeneity, the frequency of maximum sensitivity is seen to vary substantially between regions. On the right is the simulated data (blue curves) and the signal intensities from the ROI (box plots). The boxes cover the first to the third quartile, the orange line is the median, and the whiskers cover the full range. (B) Simulations and measurements showing the effect of $T_{\text{SL}}$ for the three α. Images for three examples $T_{\text{SL}}$ are shown on the left, and the simulation (blue curve) and ROI intensities (box plots) data are presented on the right.

The white contours in the Larmor frequency maps outline the regions where the local Larmor frequency is 100 ± 1 Hz. The red and blue clusters of voxels are the overlapping regions where the local Larmor frequency is 100 ± 1 Hz for all three maps. The measurement data used for comparison with the simulations were taken from the blue region.

### 4.2 Verifying resonance

The measured $S_{Bz}$ seen in Figure 4 shows clear spatial variation caused by a field‐dependent Larmor frequency and a varying frequency specificity for different values of α. The strongest signal occurs for $\omega_{z}/2\pi$ = 100 Hz in all three conditions, validating the resonance condition. The data from the ROI is presented in the box plots in Figure 4 together with simulations. This further validates the simulations and shows that the strongest signal is obtained for $\alpha =90^{\circ }$, and it decreases as α decreases. The frequency specificity is reduced for lower α.

This signal $S_{Bz}$ is proportional to the amplitude of the original images, $I_{\text{even}}$ and $I_{\text{odd}}$. This leads to a stronger signal at the bottom of the phantom that is close to a receive coil. This can also be seen where $\omega_{z}/2\pi$ = 90 Hz and 110 Hz for α = $90^{\circ }$ and $45^{\circ }$, where $S_{Bz}$ is strong for $\omega_{z}/2\pi$ = 90 Hz due to the volume of sensitivity to 90 Hz being close to receive coil elements.

### 4.3 Spin‐lock duration

Figure 4B shows the signal strength for varying $T_{\text{SL}}$. There is a good correspondence between simulations and measurements with only a slight deviation for long $T_{\text{SL}}$ and $\alpha =5^{\circ }$. The optimal $T_{\text{SL}}$ for maximum signal strength is increased for lower values of α, likely caused by a longer $T_{1\rho }$ time with increased detuning.

## 5 DISCUSSION

The key difference from other spin‐lock sequences used for detecting weak oscillating magnetic fields is the combination of excitation by spin‐lock‐inducing pulses without a pre‐excitation pulse that aligns the magnetization along the spin‐lock axis, and the use of off‐resonance spin‐lock preparation. Applying a spin‐lock pulse without a preceding excitation pulse may appear to contradict the spin‐lock concept. However, considering that the spin orientation distribution is near‐isotropic, it is clear that spin‐locking and rotating frame dynamics can be discussed independently of preceding excitation. An alternative description of the techniques used involves two‐photon excitation, which is a special case of multi‐photon excitation. Multi‐photon excitation has previously been used in NMR^32^ and recently been introduced in MRI.^33^ In contrast to standard single‐photon excitation and similar to REX, two‐photon excitation makes use of an RF pulse as well as an additional oscillatory field parallel to the $B_{0}$ field. Contrary to REX, where the off‐resonance detuning is usually kept to a minimum, in two‐photon excitation $\Delta \omega_{\text{rf}}$ is much larger than $\gamma B_{1}$ and two‐photon excitation may occur if

(4) $$\gamma B_{0}=\omega_{\text{rf}}\pm \omega_{z}\Rightarrow \omega_{z}=\pm \Delta \omega_{\text{rf}}.$$

However, this is an approximation and requires a Bloch‐Siegert shift correction (see Han and Liu^33^). The Bloch‐Siegert correction compensates for the difference between $\Delta \omega_{\text{rf}}$ and $\gamma |B_{\text{eff}}|$ as seen in Figure 1B. The frequency $\omega_{z}$ that results in an excitation can thus be calculated in the same way as the Larmor frequency in the rotating frame in Equation (1). This shows that the frequency that causes excitation in REX and two‐photon MRI is the same, determined by the precession frequency of M around $B_{\text{eff}}$ in the rotating frame. The preparation used in this work, with no preceding excitation pulse and with off‐resonance preparation pulses, is closely related to multi‐photon excitation, but the close relation to other spin‐locking techniques is less evident using the multi‐photon description.

Using the new MRI sequence, we have shown that off‐resonance RF pulses can be used to increase the Larmor frequency in the rotating frame and thereby the sensitivity to fields oscillating at this frequency. The approach was validated by comparing measurements and simulations. This increases the frequency of measurable field oscillations during spin‐lock preparation pulses without increasing SAR.

In agreement with the theoretical derivation by Han and Liu,^33^ ignoring relaxation, the excitation efficiency for multi‐photon excitation, and thereby also REX, is proportional to the strength of the $B_{1}$ field as shown in Figure 1C. The highest efficiency is thus achieved with on‐resonance preparation pulses and decreases as the detuning increases. This has the downside that lower sensitivity is achieved for high‐frequency measurements when SAR is a limiting factor and the off‐resonance frequency is increased to reduce the $B_{1}$ field strength. However, the effect of relaxation on the spin dynamics varies with the detuning, which results in practically no decrease of sensitivity for $\alpha =45^{\circ }$ compared to $\alpha =90^{\circ }$ where the $B_{1}$ is 30% lower (see Figure 4). Furthermore, the spatial variation of the Larmor frequency in the rotating frame is reduced for $\alpha =45^{\circ }$, resulting in a more homogeneous excitation. It might thus be preferable to use off‐resonance pulses, even without SAR limitations, to increase the robustness to field inhomogeneities. However, this should be verified for human brain data, since the $B_{0}$ inhomogeneity is worse than for a spherical phantom.

Using KomaMRI,^27^ we conducted the simulations in the standard frame rotating near the $B_{0}$ Larmor frequency around the direction of the static field with $T_{1}$ and $T_{2}$ relaxation times. Spin‐lock experiments should preferably include the $T_{1\rho }$ and $T_{2\rho }$ rotating frame relaxation times during the relatively long spin‐lock pulses. These describe relaxation along $B_{\text{eff}}$ and orthogonal to this, and they depend differently on molecular motion than $T_{1}$ and $T_{2}$. When $\alpha ≃0^{\circ }$ (large detuning), $T_{1\rho }≃T_{1}$, and $T_{2\rho }≃T_{2}$. When $\alpha ≃90^{\circ }$, both are similar to $T_{2}$ but longer. The changed relaxation times during RF pulses can be included in simulations, but they are unknown to us for the phantom, and vary with both spin‐lock amplitude and detuning.^25^ The approximate simulation may be a reason for the slight discrepancies with measurements, but those do not affect the conclusions, as the resonance frequencies are largely unaffected by relaxation that is slow compared to the period of oscillation. Rotating‐frame relaxation rates could used in open source MR simulators such as KomaMRI to facilitate more accurate and reproducible simulations of spin‐lock experiments.

The optimal sequence parameters, such as the angle of $B_{\text{eff}}$ (α), and spin‐lock duration ($T_{\text{SL}}$), depend on the application and are determined by, e.g., the frequency and tissue parameters of interest.

We used an MR‐compatible current‐controlled neurostimulator—otherwise used for transcranial electric stimulation and fMRI studies or MRCDI—in combination with an Arduino microcontroller to create the time‐varying magnetic field to generate the REX effect. For brain stimulation methods such as tumor‐treating fields or temporal interference stimulation, a stimulator is also needed. However, an alternative method to generate a time‐varying field for testing and development is to use the gradient system of the MR scanner.^7^

Although we have here measured the influence of the current‐induced magnetic field on the spin dynamics, we have not quantified the field from the MR measurements. To determine the density of injected currents, e.g., the magnetic field has to be quantified, which is complicated by field inhomogeneity and unknown rotating frame relaxation rates, and is left for future work. Magnetization transfer and chemical exchange saturation transfer can also cause issues for quantification when using off‐resonance irradiation.

This work has the potential to improve brain stimulation techniques. Current methods for mapping current‐induced magnetic fields, such as Magnetic Resonance Current Density Imaging (MRCDI), are limited in their frequency range. Our proposed method provides a noninvasive measure of oscillatory fields over a broader frequency range. This may be particularly beneficial for techniques such as deep brain stimulation, temporal interference stimulation, and tumor‐treating fields, which operate over a wide range of frequencies. In vivo studies are needed to evaluate the method in a clinical setting. While this study broadens the measurable frequency range, sensitivity improvements for weak natural neuronal current detection remains a separate challenge.

## 6 CONCLUSIONS

We have shown here that off‐resonance spin‐lock pulses can be used to increase the Larmor frequency in the rotating frame, which increases the measurable frequency of oscillatory fields without increasing SAR and $B_{1}$. This potentially enables mapping of the current densities that result from high‐frequency brain stimulation methods, such as temporal interference stimulation or tumor‐treating fields.

## CONFLICT OF INTEREST STATEMENT

The authors declare no potential conflict of interest.
